# Advanced Age Is Associated With Catatonia in Critical Illness: Results From the Delirium and Catatonia Prospective Cohort Investigation

**DOI:** 10.3389/fpsyt.2021.673166

**Published:** 2021-11-19

**Authors:** Jennifer Connell, Ahra Kim, Nathan E. Brummel, Mayur B. Patel, Simon N. Vandekar, Pratik Pandharipande, Robert S. Dittus, Stephan Heckers, E. Wes Ely, Jo Ellen Wilson

**Affiliations:** ^1^Critical Illness, Brain Dysfunction, and Survivorship Center, Center for Health Services Research, Nashville, TN, United States; ^2^Vanderbilt University School of Medicine, Nashville, TN, United States; ^3^Department of Biostatistics, Vanderbilt University Medical Center, Nashville, TN, United States; ^4^Division of Pulmonary, Critical Care, and Sleep Medicine, The Ohio State University Wexner Medical Center, Columbus, OH, United States; ^5^Division of Trauma and Surgical Critical Care, Departments of Surgery, Neurosurgery, and Hearing and Speech Sciences, Section of Surgical Sciences, Vanderbilt Brain Institute, Vanderbilt University Medical Center, Nashville, TN, United States; ^6^Geriatric Research, Education, and Clinical Center Service, Veterans Affairs Tennessee Valley Healthcare System, Nashville, TN, United States; ^7^Division of Anesthesiology Critical Care, Department of Anesthesiology, Vanderbilt University Medical Center, Nashville, TN, United States; ^8^Department of Psychiatry and Behavioral Sciences, Vanderbilt University Medical Center, Nashville, TN, United States; ^9^Division of Allergy, Pulmonary and Critical Care, Department of Medicine, Vanderbilt University Medical Center, Nashville, TN, United States

**Keywords:** age, catatonia, delirium, coma, critical illness

## Abstract

**Introduction:** Catatonia, characterized by motor, behavioral and affective abnormalities, frequently co-occurs with delirium during critical illness. Advanced age is a known risk factor for development of delirium. However, the association between age and catatonia has not been described. We aim to describe the occurrence of catatonia, delirium, and coma by age group in a critically ill, adult population.

**Design:** Convenience cohort, nested within two clinical trials and two observational cohort studies.

**Setting:** Intensive care units in an academic medical center in Nashville, TN.

**Patients:** 378 critically ill adult patients on mechanical ventilation and/or vasopressors.

**Measurements and Main Results:** Patients were assessed for catatonia, delirium, and coma by independent and blinded personnel, the Bush Francis Catatonia Rating Scale, the Confusion Assessment Method for the Intensive Care Unit (ICU) and the Richmond Agitation and Sedation Scale. Of 378 patients, 23% met diagnostic criteria for catatonia, 66% experienced delirium, and 52% experienced coma during the period of observation. There was no relationship found between age and catatonia severity or age and presence of specific catatonia items. The prevalence of catatonia was strongly associated with age in the setting of critical illness (*p* < 0.05). Delirium and comas' association with age was limited to the setting of catatonia.

**Conclusion:** Given the significant relationship between age and catatonia independent of coma and delirium status, these data demonstrate catatonia's association with advanced age in the setting of critical illness. Future studies can explore the causative factors for this association and further elucidate the risk factors for acute brain dysfunction across the age spectrum.

## Introduction

Catatonia, a psychomotor syndrome with motor, behavioral and affective abnormalities, was previously thought to represent a subtype of schizophrenia (1). However, it is more likely a sign of other clinical diagnoses, including mood disorders, medical including neurologic conditions, and critical illness. Catatonia occurs in up to a third of adult patients experiencing a critical illness (2, 3) with higher estimates in those already experiencing an episode of delirium (3). The prevalence of catatonia in comatose individuals is not established.

The risk factors for delirium, which is a more well-understood form of acute brain dysfunction, have previously been described. These risk factors include predisposing risk factors such as cognitive impairment, cardiovascular and renal disease, visual and hearing impairment, as well as precipitating risk factors such as acute medial illness, trauma, and medication effects (4). Advanced age is a well-known predisposing risk factor for delirium (4–6). Predisposing risk factors for the development of catatonia are not well-described, but may include genetic variants relating to neuronal structure as well as alterations in activation and connectivity of cerebral motor circuits (7). Describing potential associations between catatonia and various risk factors is a first step in better understanding the predisposing and precipitating factors, provide potential insights into pathophysiology, and better target screening and treatment. However, much work is still needed to study this topic more substantially.

To the best of our knowledge, no previous studies have explored whether advanced age is associated with catatonia prevalence in any setting. To best target screening and future treatment interventions for catatonia, we must first understand whether catatonia differentially affects a particular population. In this study we aim to understand whether the occurrence of catatonia, delirium and coma differ by age group in a critically ill adult population. We hypothesize that there is a differential effect of age on the occurrence of catatonia, delirium, and coma during critical illness.

## Methods

The Delirium and Catatonia (DeCat) Prospective Cohort Investigation is a single-center convenience sample, prospective observational cohort study nested within four NIH sponsored investigations (two clinical trials and two observational cohort studies). All parent studies enrolled critically ill patients on mechanical ventilation and/or vasopressors. Eligible participants were 18 years of age or older at the time of enrollment. The study objectives undertaken in this cohort investigation were completely novel and distinct from parent study objectives and outcomes. For this analysis, data were collected between December 2013 and December 2018. The Institutional Review Board at Vanderbilt University Medical Center (Nashville, Tennessee) approved this study. If the patient was unable to provide consent, informed consent was obtained from the patient or surrogate decision maker. At a later point, patients were reassessed as to whether they regained capacity to give consent; if capacity was demonstrated, patients were given the opportunity to give or decline further consent.

### Assessment of Catatonia, Delirium, and Coma

#### Catatonia

Catatonia signs and symptoms were measured up to twice daily by trained study personnel. Catatonia was assessed using the Bush Francis Catatonia Rating Scale (8–10) a 23-item rating scale evaluating observed characteristics, physical exam maneuvers, interview of patient, family, and caregivers, as well as review of vital signs. Although delirium cannot be evaluated in the setting of coma, many features of catatonia can still be assessed even during coma. Regardless of Richmond Agitation and Sedation Scale (RASS) score, patients were evaluated for catatonia using items from the BFCRS that can still be assessed in the setting of coma (e.g., rigidity, negativism, grasp reflex, etc.). To avoid over diagnosing catatonia in the context of critical illness, catatonic features which might be secondary to known coma or critical illness phenotype (e.g., stupor, mundane posturing, withdrawal, or mutism, etc.) were considered unable to assess on a case-by-case basis by trained study personnel and did not contribute to the diagnostic threshold for catatonia and were not rated according to symptom prevalence. Using a previously described algorithm, we converted the BFCRS score into a binary DSM-5 determination of catatonia absence or presence (3, 11). We chose to use the BFCRS to screen for the widest breadth of catatonia signs possible but chose to use the DSM-5 criterion A (>3 of 12 signs) as the reference standard for catatonia's absence or presence.

#### Delirium and Coma

Delirium was assessed up to twice daily in the ICU and daily on the medical floor until discharge using the Confusion Assessment Method for the ICU (CAM-ICU). All delirium assessment were completed by trained study personnel who were blinded to catatonia assessments. Preceding the CAM-ICU, the (RASS) was performed to assess the patient's level of arousal (12, 13). Regardless of the clinical phenotype (i.e., Hypoxemic, septic, sedative exposure) delirium was assessed in using the same methodology (14). A RASS score of −4 (arousable to physical stimulation only) or −5 (unarousable to physical stimuli) indicated the presence of coma. Symptoms of catatonia were evaluated using the BFCRS regardless of underlying delirium or coma status. Not all features of catatonia were able to be evaluated in all patients, as features of catatonia were evaluated on a case-by-case basis and individual features that were unable to be evaluated (e.g., mutism in someone on mechanical ventilation, or immobility in someone on paralytics) were not evaluated and marked as “unable to assess.” Individual features of immobility/stupor were evaluated and rated on the BFCRS regardless of underlying coma or delirium status as we were careful to not make judgements about the cause of immobility or stupor, etc. and simply described the result of the assessment.

### Study Outcomes

We were interested in understanding whether catatonia, delirium, and coma differed by age. Additionally, we sought to describe differences in presentation of catatonia between age based on presence or absence of BFCRS symptoms.

### Statistical Analysis

Descriptive statistics are presented as medians with interquartile ranges (IQR) for continuous variables and frequencies (proportions) for categorical variables. In order to understand how catatonia signs and presence differed by age, we divided the cohort into quartiles of age, rounded to the next whole number. We assessed the distribution of patients across fixed categories of brain state: ever catatonic, ever delirious, ever both catatonic and delirious, ever comatose, ever both comatose and catatonic. To examine the association between age and abnormal brain states, we used a chi-square test for trend in proportions performed for each brain state across the quartiles of age.

We used Spearman's correlation to assess correlation between BFCRS criteria to understand whether there is a linear relationship between BFCRS criteria. We generated a cluster dendogram to depict the shared variance or overlap between two BFCRS criteria using Spearman's ρ^2^ (Supplementary Figure 1) We modeled the association between age and each individual BFCRS item using an ordinal cumulative probability model for variables with more than two levels or logistic regression for binary variables. We analyzed BFCRS items with no more than 95% of observations in one category to ensure adequate number of observations in each age quartile. Robust standard errors were obtained to account for correlation in repeated assessments within individuals. All analyses were performed using statistical software R version 4.0.3 (R Foundation for Statistical Computing, Vienna, Austria; http://www.R-project.org/).

## Results

### Patient Population

We enrolled 378 critically ill adult patients between December 2013 and December 2018. Patients were separated into quartiles of age: 18–46 (*N* = 90), >46–58 (*N* = 99), >58–66 (*N* = 90), and > 66 years (*N* = 99). The cohort was 61% male, 12% were non-White/Caucasian (Table 1). The median (IQR) Sequential Organ Failure Assessment (SOFA) score at enrolment was 9.5 (7–12) and Charlson comorbidity index was 1 (0–3). This was a non-cognitively impaired population at baseline, as evidenced by the median score (IQR) of 3.0 (3.0–3.2) on the short-form for the Informant Questionnaire on Cognitive Decline in the Elderly (IQCODE). Rates of admission by primary diagnosis and admitting unit are displayed in Table 1.

**Table 1:** Baseline characteristics and brain state by age.

**Variable N (%/IQR)**	**Age**				
	**18–46**	**46–58**	**58–66**	**>66**	**Overall**
	**(*n* = 90)**	**(*n* = 99)**	**(*n* = 90)**	**(*n* = 99)**	**(*n* = 378)**
Gender (%)					
Male	60 (67%)	62 (63%)	46 (51%)	61 (62%)	229 (61%)
Female	30 (33%)	37 (37%)	44 (49%)	29 (38%)	149 (39%)
Race (%)					
White/caucasian	75 (83%)	91 (92%)	80 (89%)	88 (89%)	334 (88%)
Other	15 (17%)	9 (8%)	10 (11%)	11 (11%)	44 (12%)
ICU type (%)					
Medical	25 (28%)	48 (49%)	38 (42%)	52 (53%)	163 (43%)
Surgical	25 (28%)	39 (40%)	39 (43%)	28 (29%)	131 (35%)
Trauma	40 (44%)	11 (11%)	13 (14%)	18 (18%)	82 (22%)
Informant questionnaire on cognitive decline in the elderly at enrollment,^a^ median (IQR)	3.0 (3.0–3.0)	3.0 (3.0–3.2)	3.1 (3.0–3.2)	3.1 (3.0–3.2)	3.0 (3.0–3.2)
Charlson score at enrollment, median (IQR)^b^	0.0 (0–1)	2.0 (0.0–3.0)	2.0 (1.0–4.0)	2.0 (1.0–4.0)	1.0 (0.0–3.0)
SOFA at enrollment, median (IQR)^c^	8.0 (5.9–10.1)	10.0 (8.0–13.0)	10.0 (7.0–12.0)	10.0 (7.0–13.0)	9.5 (7.0–12.0)
Modified SOFA at enrollment, median (IQR)^d^	5.0 (3.0–7.1)	7.0 (5.0–10.0)	7.0 (5.0–10.0)	7.0 (5.0–10.0)	6.0 (4.0–9.0)
Primary reason for ICU admission, %					
Airway/COPD/asthma/PE/DVT	5 (6%)	11 (11%)	15 (17%)	17 (17%)	48 (13%)
ALI/ARDS	3 (3%)	10 (10%)	5 (6%)	10 (10%)	28 (7%)
Cardiac	2 (2%)	4 (4%)	5 (6%)	4 (4%)	15 (4%)
GI bleed/cirrhosis/pancreatitis	2 (2%)	3 (3%)	3 (3%)	2 (2%)	10 (3%)
Malignancy	0 (0%	1 (1%)	2 (2%)	1 (1%)	4 (1%)
Metabolic endocrine/renal failure	0 (0%)	3 (3%)	2 (2%)	1 (1%)	6 (2%)
Other	2 (2%)	2 (2%)	3 (3%)	1 (1%)	8 (2%)
Pneumonia/other infectious	8 (9%)	11 (11%)	7 (8%)	10 (10%)	36 (10%)
Seizures/neurological	4 (4%)	1 (1%)	0 (0%)	2 (2%)	7 (2%)
Sepsis/septic shock	14 (16%)	29 (30%)	23 (26%)	24 (24%)	90 (24%)
Surgery	7 (8%)	12 (12%)	11 (12%)	8 (8%)	38 (10%)
Trauma	42 (47%)	11 (11%)	14 (16%)	18 (18%)	85 (23%)

*IQR, interquartile range*.

a *The Short Informant Questionnaire on Cognitive Decline in the Elderly ranges from 1 to 5, with a score of 3 indicating no change in cognition over the past 10 yr, a score <3 indicating improvement, and a score >3 indicating decline in cognition, as compared with 10 yr before. A score of >3.6 indicates pre-existing cognitive impairment*.

b *Scores on the Charlson comorbidity index range from 0 to 33, with higher scores indicating a greater burden of illness; a score of 1 or 2 is associated with a mortality of ~25% at 10 yr*.

c *Scores on the Sequential Organ Failure Assessment (SOFA) range from 0 to 24 (from 0 to 4 for each of six organ systems), with higher scores indicating more severe organ dysfunction*.

d *We also used a modified SOFA score, which excluded the Glasgow Coma Scale components, because coma assessment was included separately in our models*.

### Epidemiology of Brain Dysfunction

Of the 378 patients, 88 (23%) ever met diagnostic criteria for catatonia using the DSM-5 criterion A, 250 (66%) ever had delirium, 197 (52%) experienced coma, and 56 (15%) experienced all three brain states (not concurrently) at some point during their stay. Throughout their hospital stay, 82 (22%) patients simultaneously experienced catatonia and delirium and 58 (15%) simultaneously experienced both catatonia and coma. Percentages of patients in each age quartile group who met criteria for catatonia diagnosis increased with age: 17, 16, 14, and 38% respectively (Table 2). Of those patients who experienced the catatonia in the ICU (88), 93.2% also experienced delirium, and 65.9% also experienced coma. Prevalence of BFCRS items by comatose/non-comatose brain state were described (Supplementary Figure 2). Of patients in the oldest age group, 100% of those who had catatonia also experienced delirium. Only four patients who experienced catatonia never experienced either delirium or coma.

**Table 2:** Trends in proportions of brain state by age.

Brain state N (%)^*^	**18–46 (*n* = 90)**	**46–58 (*n* = 99)**	**58–66 (*n* = 90)**	**>66 (*n* = 99)**	**Overall (*n* = 378)**	**Chi^**2**^**	***p***
Ever delirium	50 (56%)	74 (75%)	54 (60%)	72 (73%)	250 (66%)	2.75	0.0974
Ever coma	44 (49%)	63 (64%)	40 (44%)	50 (51%)	197 (52%)	0.44	0.5078
Ever catatonia	17 (19%)	18 (18%)	15 (17%)	38 (38%)	88 (23%)	9.23	0.0024
Ever both catatonia and delirium	15 (17%)	16 (16%)	13 (14%)	38 (38%)	82 (22%)	12.01	0.0005
Ever both catatonia and coma	11 (12%)	13 (13%)	11 (12%)	23 (23%)	58 (15%)	3.98	0.0461

* *Delirium was screened for with the Confusion Assessment Method for the ICU. The presence of coma was determined by a −4 (deep sedation) or −5 (unarousable) on the Richmond Agitation and Sedation Scale. Catatonia was diagnosed using >3 DSM 5 criterion A items present. Chi-squared trend tests quantified the association between the prevalence of each brain state and age category. Categories of brain state were non-mutually exclusive, meaning that patients could fall into more than one category. For example, a patient who experienced delirium and catatonia during their ICU stay would be represented in the “Ever delirium,” “Ever catatonia,” and “Ever both catatonia and delirium” rows*.

***Clinical Interpretation:** There was a significant positive correlation between prevalence of catatonia and age independent of comorbid acute brain dysfunction (delirium or coma). Of note, delirium and coma alone were not significantly correlated with age. The association between delirium and catatonia with age was only seen in the setting of catatonia*.

### Relationship Between Age and Brain State

The prevalence of catatonia (*p* = 0.0024), catatonia and delirium (*p* = 0.0005) and catatonia and coma (*p* = 0.046) increased across quartiles of age (Table 2). The association between catatonia prevalence and age was independent of delirium and coma status. The association between delirium and coma was only seen in the setting of catatonia.

### Characteristics of Catatonia by Age

We conducted a total of 1,514 assessments of catatonia using the BFCRS (Table 3). Median number of catatonia signs (when score was >0) were 3 (IQR, 1–4). The most frequently occurring catatonic signs aside from autonomic abnormalities, were immobility (41%), posturing (38%), and staring (33%) (Supplementary Table 1). This trend was consistent in comatose patients as well. In both ordinal cumulative probability models and binary logistic regression models, there was no association between age quartile and BFCRS items (Supplementary Tables 2, 3). We excluded the following items because they had too few observations: grimacing, echopraxia, mannerisms, verbigeration, waxy flexibility, Mitgehen, and combativeness.

**Table 3:** Frequency and distribution of catatonic signs by age.

**Variable^** 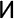 **^**	**18 to 46 (*n* = 387)**	**46+ to 58 (*n* = 399)**	**58+ to 66 (*n* = 340)**	**>66 (*n* = 388)**	**Overall (*n* = 1,514)**
Number of BFCRS symptoms present (score >0), median (IQR)	3 (1–4)	3 (1–4)	2 (1–4)	3 (1–5)	3 (1–4)
BFCRS total score, median (IQR)	6 (3–9)	5 (2–9)	4 (2–7)	6 (3–9)	5 (3–9)
Number of BFCSI symptoms present (score >0), median (IQR)	2 (0–3)	2 (0–3)	1 (0–2)	2 (0–3)	2 (0–3)
BFCSI total score, median (IQR)	2 (0–5)	2 (0–5)	1 (0–4)	1 (0–4)	2 (0–5)

^
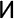
^*Catatonic signs were measured using the Bush Francis Catatonia Rating Scale*.

*Differential age quartiles did not demonstrate differences in BFCRS total score or catatonia severity (as measured by BFCSI)*.

### Clustering of BFCRS Items

**Figure 1:**
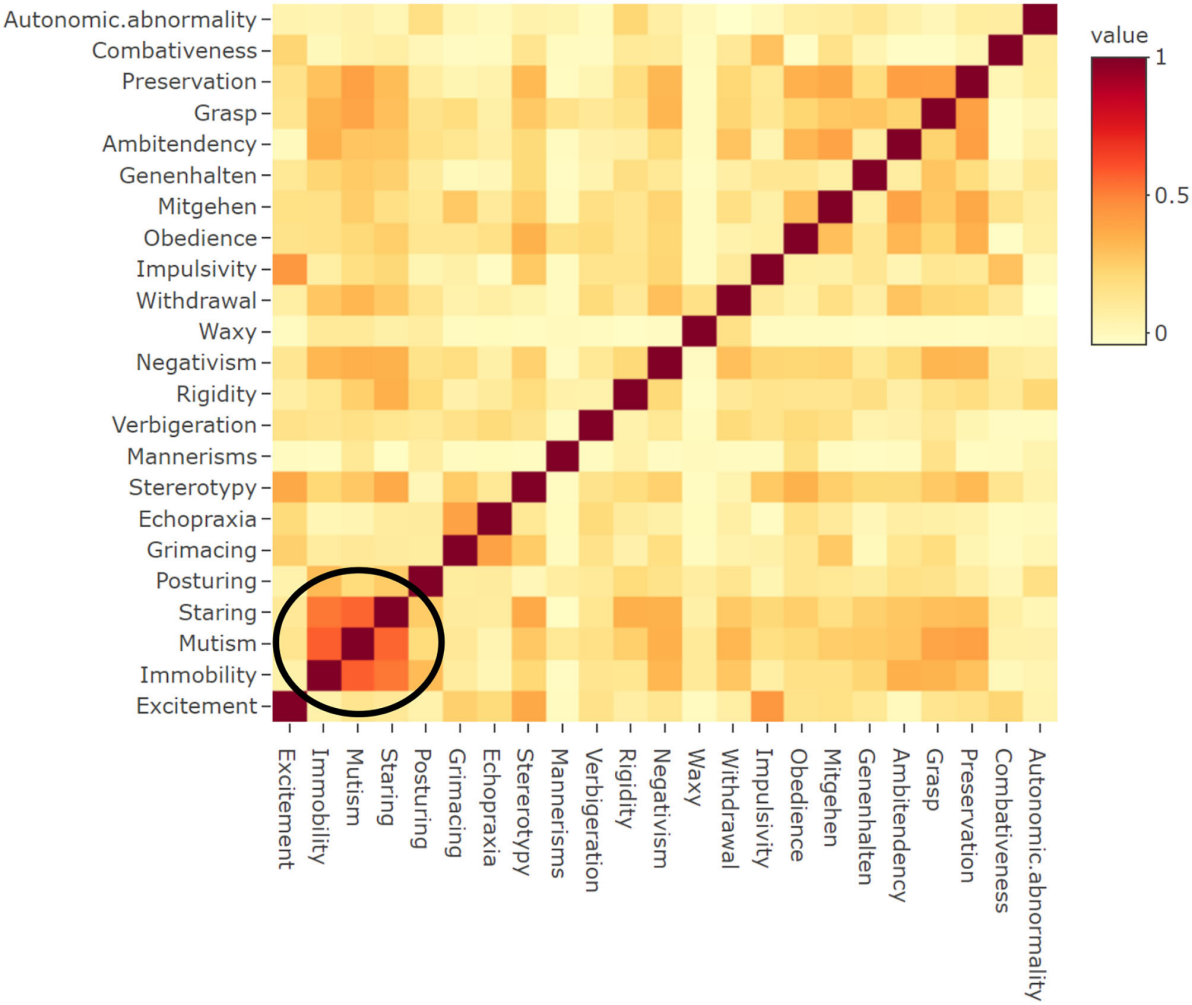
Correlation between BFCRS items. Heatmap showing correlation between items on BFCRS (*n* = 738). ^*a*^The heatmap above visually depicts the correlation between individual Bush Francis Catatonia Rating Scale (BFCRS) items in this critically ill cohort. There were moderate, positive correlations between mutism and immobility (ρ = 0.581), staring and mutism (ρ = 0.568), as well as staring and immobility (ρ = 0.529). The encircled region emphasizes the BFCRS items with highest correlations. This region demonstrates the primarily hypoactive phenotype of catatonia in the context of critical illness when delirium and coma are highly comorbid.

We examined the relationship between paired items in the BFCRS using Spearman's correlation and created a heatmap displaying the results (Figure 1). Only records with complete entries in all items were included (*n* = 738). There were moderate, positive correlations between mutism and immobility (ρ = 0.581), staring and mutism (ρ = 0.568), as well as staring and immobility (ρ = 0.529).

## Discussion

Catatonia is an under-recognized form of acute brain dysfunction in critically ill patients, despite its frequent co-occurrence with more well-described forms of brain dysfunction, such as delirium. In our study, nearly a quarter of all patients met criteria for catatonia. The presence of catatonia, co-occurrence of catatonia and delirium as well as the co-occurrence of catatonia and coma all increased with increasing age. In the oldest age group, 100% of those who had catatonia were also delirious and 60.5% also experienced coma. Also, over half of those in the older age group who experienced delirium also experienced catatonia. While this prevalence of catatonia may seem high, this is actually consistent with literature in non-critically ill and critically ill patients showing that about a third of medically ill patients experience catatonia (2, 3).

To the best of our knowledge, there have been no previous investigations exploring the prevalence of catatonia across different age groups. Our study showed that there is a significant correlation between increasing age and prevalence of catatonia. Notably, the positive association between delirium and coma with age was only seen in the setting of catatonia. Also, when catatonia occurs in the context of critical illness, it almost exclusively occurs alongside other forms of acute brain dysfunction. This may suggest that older patients should be screened more often for development of catatonia, especially in the settings of coma and delirium. Existing programs evaluating for the presence of delirium and / or coma, may consider the addition of catatonia screening for patients who experience prolonged episodes of delirium or coma, or in those with atypical psychomotor features that may suggest catatonia (e.g., increased tone, mutism in a non-mechanically ventilated patient, new grasp reflex, etc.).

The increasing prevalence of catatonia in the elderly may be due to the structural and chemical changes that occur in the brain as a result of aging. Through recent neuroimaging studies, catatonia has been associated with abnormal neuronal activity in the motor cortices, reduced GABA-A receptor density, and aberrant connectivity within the motor circuit (7, 15–17). Elderly patients have increased rates of baseline cerebral dysfunction as a result of a variety of age-related pathologies such as microvascular disease, dementia, neurotransmitter levels, and development of white matter lesions (18). Reduction in dopamine levels of ~10% per decade after entering adulthood have been associated with declining motor performance (19). Further studies are needed to better elucidate the specific contributions of various potential mechanisms of age-related brain dysfunction on development of catatonia.

The evaluation of co-occurring catatonia and coma was a novel aspect of this study. To the best of our knowledge, this represents the first report of the co-occurrence of these forms of acute brain dysfunction in the literature. These data illustrate that 65.91% of all patients with catatonia also experienced coma. Of those who experienced catatonia, 93.2% also experienced delirium. It should be noted that while delirium is an assessment of aspects of cognition (e.g., attention), and therefore cannot be examined in coma, many features of catatonia can still be assessed for in a critically-ill patient, regardless of their level of arousal.

The strikingly high rate of co-occurrence between catatonia, delirium, and coma also prompts re-evaluation of the conceptual model of acute brain dysfunction. Current models posit delirium and coma on a spectrum of dysfunction of cognition and arousal (20, 21). However, these data support recent calls to add catatonia to the conceptual framework of critical illness brain injury, with features overlapping with both delirium and coma (22). Given the vast differences in prevention and management of these conditions, accurate recognition and labeling of these syndromes is critical. Future studies should further delineate the potentially multi-dimensional model of acute brain dysfunction in critical illness that incorporates catatonia, delirium, and coma.

Current clinical practice guidelines for ICU patients recommend using available screening tools to monitor for delirium, however there is a gap in practice guidelines as no specific screening programs for catatonia exist (23, 24). Given the limited resources available in the healthcare system, screening and treatment guidelines that effectively target populations most at risk for development of catatonia are needed. Description of risk and prognostic factors are necessary for development of these guidelines.

In addition to better targeting distribution of screening, it is also necessary to understand differences in clinical presentation of catatonia between populations, as catatonia is a heterogeneous clinical entity with wide range of clinical presentations among different patients. Although the prevalence of catatonia was different across age groups, there was no association between age and the number of BFCRS signs present nor the severity score in an analysis of 1,514 separate clinical catatonia assessments.

Analysis of individual BFCRS items also revealed no association between age and presence of specific catatonic symptoms. Our data suggests that although age may be associated with catatonia, it does not appear to alter dramatically the phenotype of the disease. However, the association between specific BFCRS items demonstrates the hypoactive phenotype of catatonia in the setting of critical illness.

The primary purpose of this investigation was to describe the association between age and catatonia prevalence and presentation. Strengths of this investigation include the large cohort of patients, wide range of critical illness, detailed assessments, and the range of age represented. A known limitation of this approach is an inability to precisely describe the causality between age and catatonia. Future investigations could build on this research by incorporating other factors known to be associated with age and brain dysfunction in a multi-factorial analysis.

## Conclusion

This investigation represents the first analysis of the association between age and prevalence of catatonia in critical illness. Catatonia had a significant positive correlation with increasing age, independent of co-occurrence of other brain states. Notably, there were no significant differences in the clinical manifestations of catatonia between age groups. For the clinician, these data represent a validation of the presence of catatonia across the lifespan and should prompt consideration of catatonia as a form of acute brain dysfunction in addition to delirium and coma. Building on this work, further studies on the likely multifactorial relationship between age and catatonia will facilitate development of a predictive model to target screening and intervention.

## Data Availability Statement

The datasets analyzed for this study are available on request to the corresponding author.

## Ethics Statement

The studies involving human participants were reviewed and approved by Vanderbilt University Institutional Review Board. The patients/participants provided their written informed consent to participate in this study.

## Author Contributions

All authors listed above have contributed substantially to the conception or design of the work, acquisition and analysis of data necessary for the work, participated in drafting or revising it for intellectual content, and given their approval to the final version of the manuscript and in doing so, agree to be accountable for the content of the work.

## Funding

JW, EE, and RD received support from the Tennessee Valley Healthcare System Geriatric Research, Education and Clinical Center (GRECC). JW and MP received support from the National Institutes of Health (NIH) R01GM120484. JW reports salary support from 1KL2TR002245. NB reports support from K76AG054864. Data were collected and stored in REDCap from NIH grant UL1 TR000445. The authors would like to acknowledge that data for this cohort was collected using support from NIH grants AG035117, HL111111, R01GM120484, and K76AG054864.

## Conflict of Interest

The authors declare that the research was conducted in the absence of any commercial or financial relationships that could be construed as a potential conflict of interest.

## Publisher's Note

All claims expressed in this article are solely those of the authors and do not necessarily represent those of their affiliated organizations, or those of the publisher, the editors and the reviewers. Any product that may be evaluated in this article, or claim that may be made by its manufacturer, is not guaranteed or endorsed by the publisher.
