# Supplementary material for: Advanced Age Is Associated With Catatonia in Critical Illness: Results From the Delirium and Catatonia Prospective Cohort Investigation
Source: Front Psychiatry. 2021 Nov 19;12:673166. doi: 10.3389/fpsyt.2021.673166 (PMC8639534; doi:10.3389/fpsyt.2021.673166)
Supplement: Supplementary file 1 [file Data_Sheet_1.zip › Age and catatonia Supplemental Figure 2.docx]

**Supplemental Figure 2**: Catatonic Sign Prevalence in Comatose/Non-Comatose Patients

Percent of catatonic signs that were present in comatose (RAAS -4 or -5) versus non comatose BFCRS assessments (N=1514). Note that all catatonic signs (shown on the Y axis) had higher frequency in comatose as opposed to non comatose patients with the exception of perseveration, stereotype, automatic obedience, ambitendency, Mitgehen, and grimacing. This demonstrates the hypoactive phenotype of catatonia in the setting of coma.
