# Supplementary material for: Advanced Age Is Associated With Catatonia in Critical Illness: Results From the Delirium and Catatonia Prospective Cohort Investigation
Source: Front Psychiatry. 2021 Nov 19;12:673166. doi: 10.3389/fpsyt.2021.673166 (PMC8639534; doi:10.3389/fpsyt.2021.673166)
Supplement: Supplementary file 1 [file Data_Sheet_1.zip › Age and catatonia Supplemental Table 1.docx]

**Supplemental Table 1**: BRCRS Item Prevalence by Age

| Variable | 18 to 46  *(n=387)* | 46+ to 58  *(n=399)* | 58+ to 66  *(n=340)* | >66  *(n=388)* | Overall  *(n=1514)* |
| --- | --- | --- | --- | --- | --- |
| BFCRS Item Prevalence, % |  |  |  |  |  |
| Excitement |  |  |  |  |  |
| 0 | 97 | 92 | 96 | 93 | 94 |
| 1 | 3 | 7 | 4 | 6 | 5 |
| 2 | 0 | 2 | 0 | 1 | 1 |
| Immobility |  |  |  |  |  |
| 0 | 56 | 57 | 69 | 55 | 59 |
| 1 | 21 | 17 | 14 | 27 | 20 |
| 2 | 13 | 13 | 9 | 12 | 12 |
| 3 | 10 | 12 | 7 | 6 | 9 |
| Mutism |  |  |  |  |  |
| 0 | 82 | 84 | 88 | 71 | 81 |
| 1 | 4 | 8 | 3 | 8 | 6 |
| 2 | 9 | 4 | 4 | 13 | 8 |
| 3 | 5 | 5 | 5 | 8 | 6 |
| Staring |  |  |  |  |  |
| 0 | 66 | 65 | 71 | 66 | 67 |
| 1 | 17 | 18 | 18 | 22 | 19 |
| 2 | 12 | 6 | 6 | 5 | 7 |
| 3 | 5 | 10 | 6 | 5 | 7 |
| Posturing |  |  |  |  |  |
| 0 | 60 | 66 | 63 | 58 | 62 |
| 1 | 18 | 11 | 16 | 16 | 15 |
| 2 | 21 | 18 | 18 | 23 | 20 |
| 3 | 1 | 5 | 3 | 2 | 3 |
| Grimacing |  |  |  |  |  |
| 0 | 98 | 98 | 98 | 97 | 98 |
| 1 | 1 | 2 | 2 | 2 | 2 |
| 2 | 1 | 0 | 0 | 0 | 0 |
| Echopraxia |  |  |  |  |  |
| 0 | 100 | 99 | 99 | 99 | 99 |
| 1 | 0 | 1 | 1 | 1 | 1 |
| Stererotypy |  |  |  |  |  |
| 0 | 93 | 95 | 96 | 93 | 94 |
| 1 | 4 | 4 | 2 | 4 | 4 |
| 2 | 3 | 1 | 2 | 3 | 2 |
| 3 | 0 | 0 | 0 | 0 | 0 |
| Mannerisms |  |  |  |  |  |
| 0 | 99 | 100 | 100 | 99 | 100 |
| 1 | 1 | 0 | 0 | 1 | 0 |
| Verbigeration |  |  |  |  |  |
| 0 | 98 | 97 | 99 | 99 | 98 |
| 1 | 1 | 2 | 1 | 1 | 1 |
| 2 | 1 | 0 | 0 | 0 | 0 |
| Rigidity |  |  |  |  |  |
| 0 | 68 | 79 | 76 | 67 | 72 |
| 1 | 25 | 16 | 19 | 23 | 21 |
| 2 | 8 | 5 | 5 | 9 | 7 |
| 3 | 0 | 0 | 0 | 1 | 0 |
| Negativism |  |  |  |  |  |
| 0 | 90 | 86 | 94 | 89 | 90 |
| 1 | 7 | 7 | 5 | 7 | 6 |
| 2 | 3 | 3 | 1 | 4 | 3 |
| 3 | 0 | 4 | 0 | 1 | 1 |
| Waxy |  |  |  |  |  |
| 0 | 100 | 100 | 100 | 100 | 100 |
| 3 | 0 | 0 | 0 | 0 | 0 |
| Withdrawal |  |  |  |  |  |
| 0 | 96 | 89 | 94 | 93 | 93 |
| 1 | 2 | 3 | 2 | 2 | 2 |
| 2 | 1 | 2 | 2 | 3 | 2 |
| 3 | 0 | 6 | 3 | 2 | 3 |
| Impulsivity |  |  |  |  |  |
| 0 | 95 | 92 | 99 | 96 | 95 |
| 1 | 4 | 6 | 1 | 3 | 4 |
| 2 | 1 | 2 | 0 | 1 | 1 |
| 3 | 0 | 0 | 0 | 0 | 0 |
| Obedience |  |  |  |  |  |
| 0 | 90 | 96 | 97 | 91 | 94 |
| 1 | 8 | 3 | 2 | 6 | 5 |
| 2 | 2 | 1 | 1 | 3 | 2 |
| Mitgehen |  |  |  |  |  |
| 0 | 98 | 98 | 97 | 96 | 97 |
| 3 | 2 | 2 | 3 | 4 | 3 |
| Gegenhalten |  |  |  |  |  |
| 0 | 85 | 86 | 80 | 87 | 84 |
| 3 | 15 | 14 | 20 | 13 | 16 |
| Ambitendency |  |  |  |  |  |
| 0 | 96 | 98 | 97 | 95 | 96 |
| 3 | 4 | 2 | 3 | 5 | 4 |
| Grasp |  |  |  |  |  |
| 0 | 88 | 90 | 93 | 89 | 90 |
| 3 | 12 | 10 | 7 | 11 | 10 |
| Preservation |  |  |  |  |  |
| 0 | 91 | 95 | 95 | 93 | 93 |
| 3 | 9 | 5 | 5 | 7 | 7 |
| Combativeness |  |  |  |  |  |
| 0 | 97 | 94 | 98 | 96 | 96 |
| 1 | 2 | 3 | 1 | 4 | 3 |
| 2 | 1 | 3 | 1 | 1 | 1 |
| Autonomic abnormality |  |  |  |  |  |
| 0 | 8 | 11 | 9 | 10 | 9 |
| 1 | 15 | 18 | 19 | 15 | 17 |
| 2 | 24 | 28 | 29 | 31 | 28 |
| 3 | 53 | 43 | 43 | 44 | 46 |
