# Supplementary material for: Advanced Age Is Associated With Catatonia in Critical Illness: Results From the Delirium and Catatonia Prospective Cohort Investigation
Source: Front Psychiatry. 2021 Nov 19;12:673166. doi: 10.3389/fpsyt.2021.673166 (PMC8639534; doi:10.3389/fpsyt.2021.673166)
Supplement: Supplementary file 1 [file Data_Sheet_1.zip › Age and catatonia Supplemental Table 2.docx]

**Supplemental Table 2:** Results of ordinal regression and binary logistic regression models of BFCRS items adjusted by age

| **BFCRS Item** | **Chi** | **p** |
| --- | --- | --- |
| Excitement | 0.18 | 0.98 |
| Immobility | 2.61 | 0.45 |
| Mutism | 2.15 | 0.54 |
| Staring | 0.59 | 0.89 |
| Posturing | 0.67 | 0.88 |
| Stererotypy | 0.07 | 0.99 |
| Rigidity | 1.74 | 0.62 |
| Negativism | 0.33 | 0.95 |
| Withdrawal | 0.25 | 0.96 |
| Impulsivity | 0.15 | 0.98 |
| Obedience | 0.25 | 0.96 |
| Autonomic abnormality | 3.01 | 0.39 |
| Gegenhalten | 1.97 | 0.57 |
| Ambitendency | 1.20 | 0.75 |
| Grasp | 1.07 | 0.78 |
| Preservation | 3.05 | 0.38 |
| Combativeness | 5.77 | 0.12 |

There was no association between individual BFCRS items and increasing age.
