# Supplementary material for: Advanced Age Is Associated With Catatonia in Critical Illness: Results From the Delirium and Catatonia Prospective Cohort Investigation
Source: Front Psychiatry. 2021 Nov 19;12:673166. doi: 10.3389/fpsyt.2021.673166 (PMC8639534; doi:10.3389/fpsyt.2021.673166)
Supplement: Supplementary file 1 [file Data_Sheet_1.zip › Age and catatonia Supplemental Table 3.docx]

**Supplemental Table 3:** Results of ordinal regression and binary logistic regression models of BFCRS items adjusted by age, comatose brain state, and delirious brain state.

| **BFCRS Item** | **Age** | | **Comatose** | | **Delirious** | |
| --- | --- | --- | --- | --- | --- | --- |
|  | **Chi** | **p** | **Chi** | **p** | **Chi** | **p** |
| Excitement | 0.18 | 0.98 | 0.16 | 0.69 | 0.03 | 0.99 |
| Immobility | 2.61 | 0.45 | 15.7 | 0.0001 | 1.476 | 0.22 |
| Mutism | 2.15 | 0.54 | 1.54 | 0.22 | 1.47 | 0.01 |
| Staring | 0.59 | 0.89 | 4.10 | 0.04 | 1.47 | 0.22 |
| Posturing | 0.67 | 0.88 | 6.07 | 0.01 | 3.4 | 0.07 |
| Stererotypy | 0.07 | 0.99 | 0.01 | 0.94 | 0.07 | 0.79 |
| Rigidity | 1.74 | 0.62 | 1.37 | 0.24 | 1.39 | 0.23 |
| Negativism | 0.33 | 0.95 | 0.37 | 0.54 | 0.08 | 0.78 |
| Withdrawal | 0.25 | 0.96 | 0.15 | 0.69 | 0.01 | 0.91 |
| Impulsivity | 0.15 | 0.98 | 0.06 | 0.80 | 0.00 | 0.99 |
| Obedience | 0.25 | 0.96 | 0.16 | 0.69 | 0.04 | 0.85 |
| Autonomic abnormality | 3.01 | 0.39 | 10.79 | 0.001 | 0.02 | 0.89 |
| Gegenhalten | 1.97 | 0.57 | 1.87 | 0.17 | 0.93 | 0.33 |
| Ambitendency | 1.20 | 0.75 | 0.27 | 0.60 | 4.82 | 0.15 |
| Grasp | 1.07 | 0.78 | 7.86 | 0.005 | 0.08 | 0.78 |
| Preservation | 3.05 | 0.38 | 0.00 | 0.97 | 8.42 | 0.004 |
| Combativeness | 5.77 | 0.12 | 9.86 | 0.002 | 2.96 | 0.09 |

There was no association between individual BFCRS items and increasing age, co-occurrence of coma, or delirium.
