# Supplementary material for: Advanced Age Is Associated With Catatonia in Critical Illness: Results From the Delirium and Catatonia Prospective Cohort Investigation
Source: Front Psychiatry. 2021 Nov 19;12:673166. doi: 10.3389/fpsyt.2021.673166 (PMC8639534; doi:10.3389/fpsyt.2021.673166)
Supplement: Supplementary file 1 [file Data_Sheet_1.zip › Catatonia and Age Supplemental Figure 1.docx]

**Supplemental Figure 1:** Dendrogram^a^ of BFCRS items.


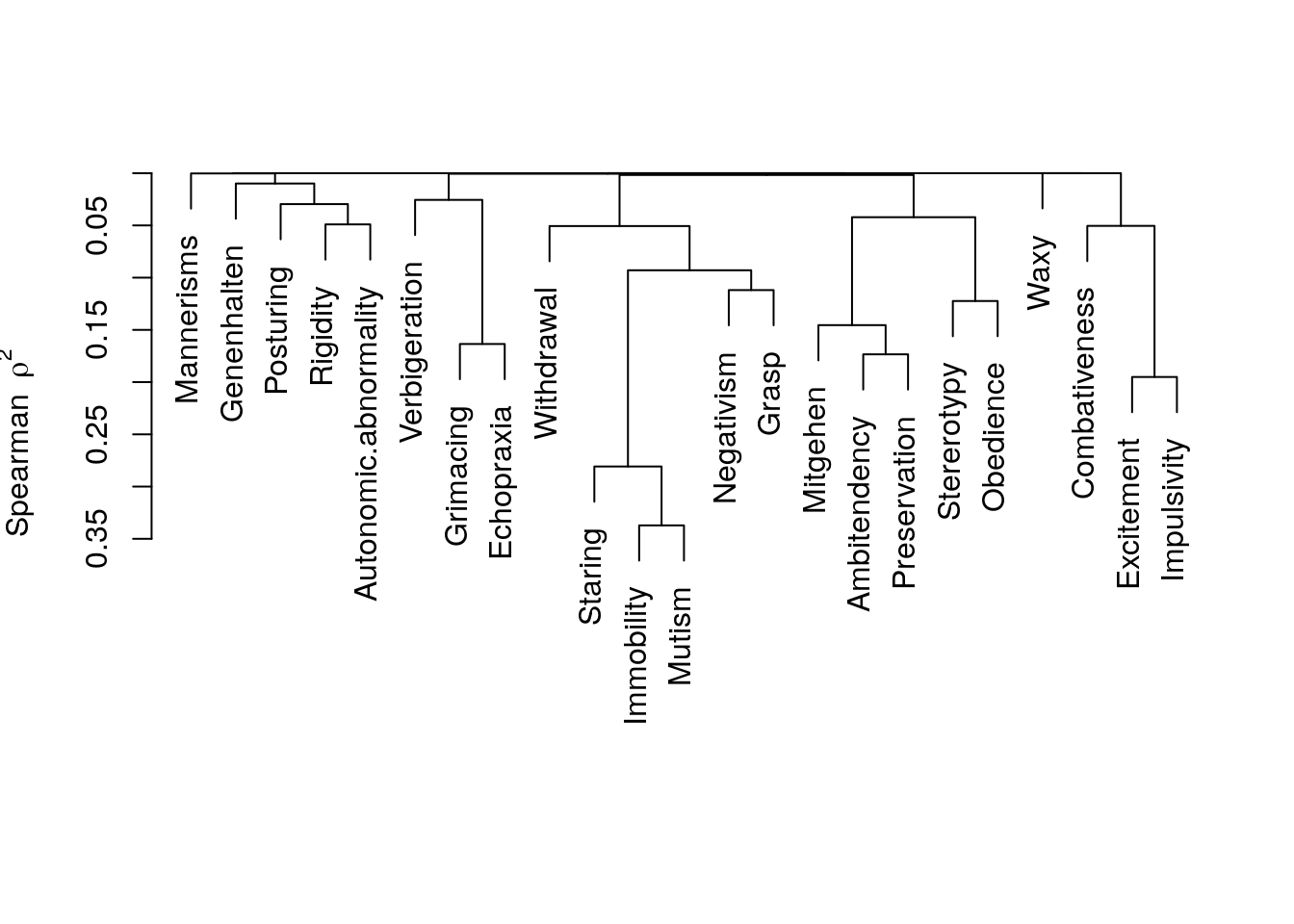


^a^Dendogram visually depicts the shared variance or overlap in individual Bush Francis Catatonia Rating Scale (BFCRS) items. Those BFCRS items with a greater Spearman p^2^ have a greater overlap or correlation with one another (e.g., immobility and mutism).
